# Ischemia as a common trigger for Alzheimer’s disease

**DOI:** 10.3389/fnagi.2022.1012779

**Published:** 2022-09-26

**Authors:** Karin Elman-Shina, Shai Efrati

**Affiliations:** ^1^Sagol Center for Hyperbaric Medicine and Research, Shamir Medical Center (Assaf Harofeh), Tzerifin, Israel; ^2^Sackler School of Medicine, Tel Aviv University, Tel Aviv, Israel; ^3^Sagol School of Neuroscience, Tel Aviv University, Tel Aviv, Israel; ^4^Research and Development Unit, Shamir Medical Center (Assaf Harofeh), Tzerifin, Israel

**Keywords:** Alzheimer’s disease, neuroinflammation, hyperbaric oxygen (HBO) therapy, brain oxygenation, cerebral ischemia, cognitive improvement

## Abstract

Alzheimer’s disease has various potential etiologies, all culminating in the accumulation of beta -amyloid derivatives and significant cognitive decline. Vascular-related pathology is one of the more frequent etiologies, especially in persons older than 65 years, as vascular risk factors are linked to both cerebrovascular disease and the development of AD. The vascular patho-mechanism includes atherosclerosis, large and small vessel arteriosclerosis, cortical and subcortical infarcts, white matter lesions, and microbleeds. These insults cause hypoperfusion, tissue ischemia, chronic inflammation, neuronal death, gliosis, cerebral atrophy, and accumulation of beta-amyloid and phosphorylated tau proteins. In preclinical studies, hyperbaric oxygen therapy has been shown to reverse brain ischemia, and thus alleviate inflammation, reverse the accumulation of beta-amyloid, induce regeneration of axonal white matter, stimulate axonal growth, promote blood–brain barrier integrity, reduce inflammatory reactions, and improve brain performance. In this perspective article we will summarize the patho-mechanisms induced by brain ischemia and their contribution to the development of AD. We will also review the potential role of interventions that aim to reverse brain ischemia, and discuss their relevance for clinical practice.

## Introduction

Alzheimer’s disease (AD) is a complex multifactorial disease with various etiologies, culminating in the accumulation of beta-amyloid protein and neurofibrillary tangles. One of the more frequent etiologies, especially in persons older than 65 years is vascular-related pathology. Vascular risk factors are tightly linked to both cerebrovascular disease and the development of AD (Custodio et al., 2017; Rost et al., 2022). Cerebral ischemia accelerates the onset of dementia by 10 years. In about 10% of individuals with dementia, its onset is soon after a first stroke; and in more than 40%, after a repeated stroke (Pluta et al., 2021b). Within 25 years after stroke, the estimated development of dementia is about 48% (Snowdon et al., 1997). Furthermore, as up to 90% of persons with AD have cerebral hypoperfusion and pathological features of amyloid angiopathy (de la Torre, 2016), it has become clear that ischemia prevention should be addressed as early as possible in order to reverse the natural history of the disease. The vascular pathology includes atherosclerosis, large and small vessel arteriosclerosis, cortical and subcortical infarcts, white matter lesions, and microbleeds. These cause hypoperfusion, tissue ischemia, chronic inflammation, neuronal death, gliosis, cerebral atrophy, and the accumulation of beta- amyloid and phosphorylated tau proteins (Snowdon et al., 1997).

In the current article we will refer to oxygen deprivation, hypoxia, as a result of ischemia. Ischemia, a condition of blood flow restriction or reduction, can lead among others to hypoxia. Hypoxia refers to a decrease in tissue oxygenation. It can result from ischemia but it can also occur in the absence of it. As for example, it can be a consequence of high altitude or exposure to inhalation of toxic gases as carbon monoxide. In this article we will review the pathophysiological processes related to hypoxia that is induced by cerebral ischemia in AD, and the interventions that have the potential to counteract them.

## Brain oxygenation and consumption

The brain constitutes only 2% of the body mass (1400 g), while it receives a large proportion (12–15%) of the resting cardiac output in the adult, 20% of the total oxygen supply, and up to 30% of total body energy consumption. Oxygen is continuously consumed by the brain at tissue oxygenation, ranging from 90 mm Hg, very close to capillaries, to less than 30 mmHg in more distal regions. Under normal healthy conditions, brain metabolism reaches the upper limit of oxygen consumption, which makes it dependent on cerebral blood flow. At each point in time, the cerebral blood flow shifts to more active regions (task-dependent) at the expense of other less active regions. The local autoregulation mechanisms that control energy demand and blood flow supply play a critical role in the adjustment of tissue partial oxygen pressure in response to dynamically varying brain activity (Masamoto and Tanishita, 2009). Accordingly, the brain is the most sensitive organ to reduced arterial oxygen pressure (PaO2). For example, reduction of PaO2 to 65 mm Hg impairs the brain’s ability to perform complex tasks. At 55 mmHg, the short-term memory is impaired, and PaO2 of 30 mmHg induces loss of consciousness (Hadanny and Efrati, 2015; Chen et al., 2020). Much has been learned from the physiological changes that occur while being in a high-altitude environment (Luks and Hackett, 2022). Climbing to an altitude of 2,500 m above sea level exposes an individual to major reduction in the barometric pressure and the oxygen in the air. These reductions lead to impairments in attention, memory, judgment, and emotional regulation. Spatial attention may be particularly affected by high-altitude exposure. At high-altitude, impairment has been observed in behavioral tests of visual attention (e.g., the digit symbol substitution test and visual search task), with slowed reaction times (Yan et al., 2011). Moreover, in a neuroimaging study, high-altitude exposure affected brain areas related to attention processing, including the occipital lobe, parietal lobe, sensory-perceptual regions, and frontoparietal attention networks (Yan et al., 2011). High-altitude exposure has also been shown to decrease accuracy in tests of verbal/visual memory, and to prolong response time in tests of visual/auditory reaction time (Zhang et al., 2017; Luks and Hackett, 2022).

## The vascular etiology of Alzheimer’s disease

**FIGURE 1:**
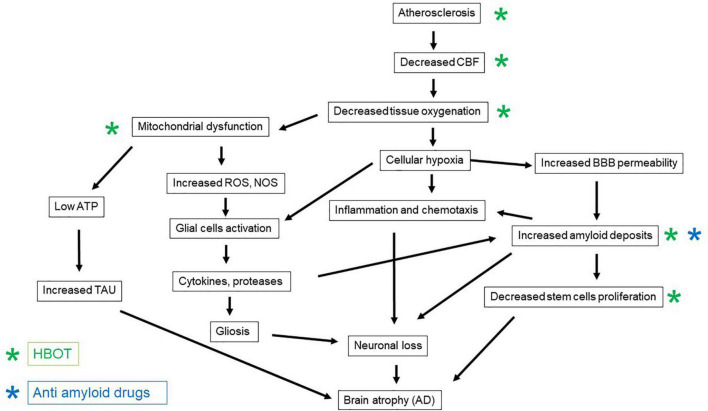
The patho-mechanisms induced by brain ischemia and the development of Alzheimer’s disease, and the counteractive effects of anti-amyloid medications and HBOT.

The vascular etiology of AD comprises vascular factors that enhance the atherosclerotic process, such as hypertension, diabetes mellitus, dyslipidemia, and obesity, together with a sedentary lifestyle (Kivipelto et al., 2005; Cunningham et al., 2020; Lee et al., 2020). The thickening of the capillary basal membrane and the accumulation of collagen in the vascular endothelium culminate in vascular atrophy of the vascular terminations (Custodio et al., 2017). These changes reduce the cerebral blood flow and as a result cerebral oxygenation (Custodio et al., 2017). The combination of cardiovascular and cerebrovascular disorders results in chronic cerebral hypoperfusion and as a result, hypoxia related inflammation, neuronal energy deprivation, and the formation of the so-called “senile plaques” and neurofibrillary tangles in the brain (Aliev et al., 2014; Figure 1). As a consequence of chronic hypoperfusion, the oxygen-deprived mitochondria generate more reactive oxygen species (ROS) per ATP and fewer mitochondria per neuronal cell (Sims and Anderson, 2002; Yang et al., 2018). The accumulation of ROS due to the mitochondrial dysfunction stimulates the expression of nitric oxide synthase (NOS) enzymes (Aliev et al., 2014). These collectively contribute to dysfunction of the blood-brain barrier (BBB) and further damage to the brain parenchyma (Aliev et al., 2014). This vicious pathophysiological cascade results in late chronic neuronal damage and loss in distant brain regions that were not directly affected during the acute insult (Pluta et al., 2021a). Moreover, post-ischemic BBB damage enables extravasations of beta-amyloid to the brain parenchyma, which further enhances the vicious pathological process (Pluta et al., 2021a).

Following ischemia, soluble beta-amyloid is delivered to the brain from the circulation, through the vascular bed; this contributes to amyloidosis, plaque accumulation, and cerebral amyloid angiopathy (further narrowing the blood vessels and reducing blood flow) (Pluta et al., 2021a; Figure 1). Two forms of vascular pathology are recognized in AD: small vessel disease and cerebral amyloid angiopathy (CAA) (Attems and Jellinger, 2014). Cerebral small vessel disease refers to a group of diseases that affect cerebral small arteries and microvessels. These can be seen as white matter hyperintensities, cerebral microbleeds, and lacunes on magnetic resonance imaging (Utter et al., 2008). Carriers of the ε4 allele of the Apolipoprotein E (APOE) gene do not efficiently break down beta-amyloid plaques; and this allele is less efficient than others in maintaining cerebral homeostasis of lipid transport, synaptic integrity, glucose metabolism, and cerebrovascular function (Utter et al., 2008). CAA is a risk factor for intracerebral hemorrhage and cognitive impairment. The relatively high prevalence of CAA among individuals with dementia has led to the supposition that CAA affects cognition through its deleterious effect on the cerebral microvasculature. However, the exact mechanisms by which CAA affects cognition are not firmly established (Reijmer et al., 2016).

## Neuroinflammation and ischemia

Ischemia induces a massive neuroinflammatory response that can be long lasting, even after reperfusion therapy, and that may result in neurodegeneration (Iadecola and Anrather, 2011; Yang et al., 2018). Microglia and astrocytes belong to the first line of defense and are activated a few minutes after an ischemic event. Activated microglia develop many characteristics of macrophages, including ameboid morphology, migratory capacity, phagocytosis, and major histocompatibility complex (MHC) class-II restricted antigen presentation (Iadecola and Anrather, 2011). Within 1 day, the influx of monocytes increases due to additional injury to the BBB by the neuroglial inflammatory factors (Pluta et al., 2021b). The neutrophils migrate to the damaged areas and release additional cytokines, proteolytic enzymes, and ROS, which initiate secondary damage to the already damaged brain tissue (Pluta et al., 2021a). The number of neutrophils following ischemia directly corresponds to the size of the brain injury. In addition to neutrophils, T and B lymphocytes, natural killer cells, mast cells, and dendritic cells infiltrate the brain and concentrate around the ischemic regions. At a later stage, the macrophages become more dominant, as they are essential for the regeneration process. This pathophysiology cascade induces additional neuronal loss, with irreversible interruption of the neuronal network, and leads to amplification of the inflammatory cycle (Pluta et al., 2021a).

## Accumulation of beta-amyloid and phosphorylated tau in response to ischemia

The amyloid protein is a neurotoxic substance that induces intracellular processes in post-ischemic neurons, astrocytes, and microglia; this further enhances neuronal and glial injury and death following ischemia (Pluta et al., 2021a). Beta-amyloid is a 38 to 43 amino acid long peptide generated by the sequential proteolytic cleavage of amyloid precursor protein (APP) by beta- and gamma-secretases. Though the exact physiological function of APP has not been identified, the over-production of beta-amyloid generated from APP is well recognized as a contributor to AD development. In addition, APP has been linked to non-effective clearance and degradation of beta-amyloid (Sengupta et al., 2016).

Following brain ischemia, beta-amyloid plaques can be seen in the hippocampus, thalamus, brain cortex, corpus callosum, and around the lateral ventricles (Pluta et al., 2021a). Moreover, clinical investigations of persons with ischemic brain injury have shown higher levels of plasma beta-amyloid and lower levels of alpha-secretase mRNA. APP cleavage *via* beta- and gamma-secretase to form beta-amyloid is known as the amyloidogenic pathway (Pluta et al., 2021a). In response to BBB damage, the influx of inflammatory cytokines, together with the soluble form of beta-amyloid, is increased (Pluta et al., 2021a; Figure 1). The burst of cytokine release further exacerbates the pathophysiological cascade (Iadecola and Anrather, 2011). For example, interleukin (IL)-1 stimulates ischemic neurons to amyloidogenic processing of APP, together with the induction of inflammatory factors (Pluta et al., 2021a).

The other hallmark of AD, the tau protein, is also found in microglia, astrocytes, and oligodendrocyte, in the hippocampus and the brain cortex, following ischemia (Pluta et al., 2021a). Hyperphosphorylation of tau dominates in neuronal cells and is concurrent with apoptosis of neurons (Pluta et al., 2021a). Tau protein was also detected in human plasma samples after ischemic brain injury, and correlates with the progression of post-ischemic neuronal damage (Pluta et al., 2021a).

Many clinical trials targeting beta-amyloid and tau proteins in various forms, including their degradation and clearance, have failed to improve the cognitive outcome. This suggests that these proteins are not the main drivers of AD (Gulisano et al., 2019; Breijyeh and Karaman, 2020). AD has been described as having multiple causes, including genetic and environmental factors, age-related events, and pathological conditions such as diabetes, traumatic brain injury, and aberrant microbiota, which affect the aggregation of beta-amyloid (Gulisano et al., 2019). White matter lesions and microhemorrhages, dyslipidemia, altered brain insulin signaling, and insulin resistance all contribute to tau and beta-amyloid pathogenesis. Furthermore, oxidative and mitochondrial damage, inflammation, and hypoperfusion serve as mechanistic links between pathophysiological features of AD and ischemia (He et al., 2020). Hence, these have become new targets for upstream modification and treatment of the disease.

## Neuronal stem cells in ischemia and Alzheimer’s disease

The brain is capable of self-repair after stroke and other insults. In response to an acute injury, brain plasticity affords function and structure reorganization at various levels, from molecular and cellular mechanisms, to changes in anatomy, neurochemistry, and the generation of new neurons by neurogenesis (Cuartero et al., 2021). Stroke has been reported to drive neural stem cells, mostly from two main brain regions: the subventricular zone of the lateral ventricles and the subgranular zone of the dentate gyrus of the hippocampus (Cuartero et al., 2021). However, despite the clear increase in neuroblast proliferation and migration after stroke, only a small proportion of immature neurons reaches the damaged area, and only about 0.2% of these immature neurons eventually fully maturate and integrate into the infarcted region (Cuartero et al., 2021). The low proportion may be due to an unsupportive ischemic environment, inflammation, and deficit of functional connections or necessary trophic support (Cuartero et al., 2021). In AD, when ischemia is involved in a repeated manner, the abnormal proliferation and maturation of neural stem cells might play a critical role in the resultant brain function, and have later consequence in cognitive decline.

In addition to the above, in AD, the aggregated beta-amyloid-42 was shown to promote accumulation of basal and ATP-triggered intracellular calcium concentrations, and to cause neuronal and progenitor cell death, as well as reduced neuronal differentiation (Waldau and Shetty, 2008; Figure 1). Moreover, increasing evidence shows that AD impairs the maturation of neuronal progenitors into neurons (Waldau and Shetty, 2008; Scopa et al., 2020).

## Interventions aimed to reverse brain ischemia in Alzheimer’s disease

Several interventions have aimed to improve and prevent ischemia-related cognitive decline. Some of these have focused on reverting and managing vascular risk factors. The FINGER study (Finnish Geriatric Intervention Study to Prevent Cognitive Impairment and Disability) demonstrated that a multidomain intervention including diet, exercise, cognitive training, and vascular risk monitoring improved cognitive functions in at-risk elderly people (Ngandu et al., 2015). Each of the cardiovascular risk factors examined was an independent risk factor for dementia. These include hypertension, smoking, obesity, hyperlipidemia, diabetes mellitus, and lack of exercise (Caprio and Sorond, 2019). Interventions, including medications and lifestyle modifications to target these risk factors, have resulted in cognitive improvement (Caprio and Sorond, 2019; Saito et al., 2019).

Among lifestyle modifications, diet and nutrition play a major role. Specific nutritional interventions such as the Mediterranean diet, the DASH (Dietary Approaches to Stop Hypertension) diet, the MIND (Mediterranean and DASH Intervention for Neurodegenerative Delay) diet, and the ketogenic diet were shown to have positive effects on prevention of AD (Saito et al., 2019) and to improve cognitive function (Rusek et al., 2019; Ballarini et al., 2021).

Several medications, such as aspirin and other an anti-platelet drugs, are in clinical use for secondary stroke prevention in patients with vascular risk factors (Judge et al., 2020). However, these have not yet been proven to affect cognitive function. Another intervention, namely carotid stenting, in asymptomatic individuals with ipsilateral ischemia and 80% stenosis, showed improved brain perfusion and cognitive function three months after the procedure (Piegza et al., 2021). In addition, the beneficial cognitive effect of revascularization holds also for symptomatic patients with carotid stenosis (Nishio et al., 2010; Chen et al., 2012, 2017; Wendell et al., 2012; Piegza et al., 2021).

A medication that is used for individuals with mild cognitive impairment (MCI) and vascular cognitive impairment is Ginkgo biloba extract (EGB761) called Cerebonin. The drug has been shown to improve cognition, behavior, and activities of daily living (ADL) both in individuals with AD and with vascular dementia, through an unknown mechanism (Kandiah et al., 2019). Importantly, anti-amyloid medications, which are monoclonal antibodies that target beta- amyloid plaques for their removal, have failed to demonstrate significant cognitive improvement (Gulisano et al., 2019; Walsh et al., 2021; Figure 1).

The tau protein has received growing attention in recent years (Congdon and Sigurdsson, 2018). In addition to beta-amyloid-targeted medications, many medications have been developed to target the tau protein. The mechanism of action of anti-tau medications may involve one of the following: inhibition of kinases, aggregation of tau, or stabilization of microtubules. Currently, the majority of the tau-targeting medications in clinical trials are immunotherapies (active and passive immunizations), which will hopefully provide significant benefit (Congdon and Sigurdsson, 2018). Unfortunately, the first tau immunization in clinical trials failed to show clinical improvement in addition to tau removal (Mullard, 2021).

Several supplements, such as curcumin, have been found to have anti-inflammatory, anti-ischemic, and anti-amyloid effects (Pluta et al., 2018). However, these supplements have not been appropriately evaluated in prospective randomized clinical trials.

## Reversal of brain ischemia by hyperbaric oxygen therapy

Hyperbaric oxygen treatment is the medical administration of 100% oxygen at environmental pressure greater than 1 atmosphere absolute (ATA). HBOT has been shown to improve neurological function and quality of life in individuals who have had a stroke, anoxic brain damage, or traumatic brain injury (Efrati et al., 2013; Hadanny et al., 2015; Tal et al., 2015; Shapira et al., 2018). Furthermore, HBOT has been shown to improve memory domains in individuals with late chronic strokes, in correlation to increased medial temporal lobe perfusion in SPECT (Boussi-Gross et al., 2015). HBOT has demonstrated significantly enhanced cognitive performance in healthy older persons (Hadanny et al., 2020). The main improved domains were attention, information processing speed, and executive function (set shifting), in addition to global cognitive functions. These findings were in correlation to enhanced brain perfusion in the superior and middle frontal gyri, the supplementary motor area, and the superior parietal lobule (Hadanny et al., 2020).

The neuroplasticity mechanisms of HBOT-induced cognitive improvements include brain angiogenesis and increased cerebral vascular flow, stem cell proliferation, regeneration of axonal white matter and axonal growth, repair of BBB integrity, and amelioration of inflammation (Efrati and Ben-Jacob, 2014; Figure 1). At the cellular level, HBOT can improve cellular metabolism, reduce apoptosis, alleviate oxidative stress, enhance mitochondrial function in neurons and glial cells, and increase levels of neurotrophins and nitric oxide (Efrati and Ben-Jacob, 2014; Figure 1). The standard protocol for induction of significant neuroplasticity consists of 60 daily sessions of 2 ATA, 100% oxygen, 90 min each session, with 5-min air breaks every 20 min. These fluctuations in oxygen concentrations from very high to normal levels have been interpreted as a relative lack of oxygen, despite the presence of extra oxygen. Hence, the fluctuations generated by HBOT can induce many of the mediators and cellular mechanisms that are usually induced in hypoxia. This is the so-called hyperoxic-hypoxic paradox (Hadanny and Efrati, 2020). The main cellular mechanisms that occur during intermittent hyperoxia include increased hypoxia inducible factor (HIF), vascular endothelial growth factor (VEGF), and sirtuin (SIRT); mitochondrial biogenesis; and stem cell proliferation and migration (Figure 1).

Until now, the effect of HBOT on AD pathology has been studied mainly in animal models, and anecdotally reported in several clinical case reports. Chen et al. (2020) evaluated the effect of HBOT on 83 individuals with AD pathology, of whom 42 had dementia, 11 had amnestic MCI, and 30 were AD controls. Neurocognitive assessment and ADL evaluation were performed at baseline and after 1, 3, and 6 months of follow up. The results showed significant improvement in Mini-Mental State Exam (MMSE) and Montreal Cognitive Assessment (MoCA) scores after 1 month of HBOT, in individuals with AD dementia and amnestic MCI, compared to the control group. The ADL score was also significantly improved after 1 and 3 months follow up in those with AD.

Our group examined the effect of HBOT on AD pathology in old 3xTg-AD mice (mice genetically induced to exhibit both beta-amyloid and tau pathology). The protocol included one group that was treated with HBOT (administration of 100% oxygen at 2 ATA; HBOT group) and a control group that was treated with normobaric air (21% oxygen at 1 ATA; control group) for 60 min daily for 14 consecutive days. Following these treatments, the mice were subjected to a battery of behavioral tasks (Y-maze, open-field test, and object recognition test). In all these tests, 3xTg mice showed impaired performance compared to non-transgenic controls, and HBOT significantly improved or restored the 3xTg-treated mouse behavior. The HBOT group had less hypoxia, beta-amyloid burden, and tau phosphorylation and the cognitive performance was improved. Furthermore, in the HBOT group, a morphological change was evident in the microglia near the plaques, to a more ramified state, the secretion of pro-inflammatory cytokines, namely interleukin (IL)-1β and tumor necrosis factor (TNF) alpha (TNF-α) was lower; and the secretion of anti-inflammatory cytokines (IL-4, IL-10) was higher (Shapira et al., 2018).

## Conclusion

Ischemia is a common trigger for several neurodegenerative diseases including AD. Ischemia contributes to chronic cerebral hypoperfusion, accumulation of beta- amyloid and tau proteins, neuroinflammation with glial activation, BBB damage, mitochondrial dysfunction, and neuronal loss. Reversal of ischemia should be a major therapeutic target in the prevention and treatment of cognitive decline related to AD.

## Data availability statement

The original contributions presented in this study are included in the article/supplementary material, further inquiries can be directed to the corresponding author.

## Author contributions

KE-S reviewed the literature and wrote the manuscript. SE reviewed and revised it. Both authors contributed to conception, design of the article, contributed to manuscript revision, read, and approved the submitted version.

## Abbreviations

CBF: cerebral blood flow
BBB: blood brain barrier
HBOT: hyperbaric oxygen therapy
ROS: reactive oxygen species
NOS: nitric oxide synthase
AD: Alzheimer’s disease.
